# Impedance-Matching Hearing in Paleozoic Reptiles: Evidence of Advanced Sensory Perception at an Early Stage of Amniote Evolution

**DOI:** 10.1371/journal.pone.0000889

**Published:** 2007-09-12

**Authors:** Johannes Müller, Linda A. Tsuji

**Affiliations:** Humboldt-Universität zu Berlin, Museum für Naturkunde, Berlin, Germany; University of Cambridge, United Kingdom

## Abstract

**Background:**

Insights into the onset of evolutionary novelties are key to the understanding of amniote origins and diversification. The possession of an impedance-matching tympanic middle ear is characteristic of all terrestrial vertebrates with a sophisticated hearing sense and an adaptively important feature of many modern terrestrial vertebrates. Whereas tympanic ears seem to have evolved multiple times within tetrapods, especially among crown-group members such as frogs, mammals, squamates, turtles, crocodiles, and birds, the presence of true tympanic ears has never been recorded in a Paleozoic amniote, suggesting they evolved fairly recently in amniote history.

**Methodology/Principal Findings:**

In the present study, we performed a morphological examination and a phylogenetic analysis of poorly known parareptiles from the Middle Permian of the Mezen River Basin in Russia. We recovered a well-supported clade that is characterized by a unique cheek morphology indicative of a tympanum stretching across large parts of the temporal region to an extent not seen in other amniotes, fossil or extant, and a braincase specialized in showing modifications clearly related to an increase in auditory function, unlike the braincase of any other Paleozoic tetrapod. In addition, we estimated the ratio of the tympanum area relative to the stapedial footplate for the basalmost taxon of the clade, which, at 23∶1, is in close correspondence to that of modern amniotes capable of efficient impedance-matching hearing.

**Conclusions/Significance:**

Using modern amniotes as analogues, the possession of an impedance-matching middle ear in these parareptiles suggests unique ecological adaptations potentially related to living in dim-light environments. More importantly, our results demonstrate that already at an early stage of amniote diversification, and prior to the Permo-Triassic extinction event, the complexity of terrestrial vertebrate ecosystems had reached a level that proved advanced sensory perception to be of notable adaptive significance.

## Introduction

The evolution of modern terrestrial ecosystems is inseparably tied to the origin and diversification of amniote vertebrates [1]. Thus, adaptively significant evolutionary novelties and the timing of their appearance within amniotes are important markers for evaluating the level of ecological complexity. Among modern amniotes, sensory perception such as impedance-matching hearing is highly important for both prey detection and intraspecific communication, requiring a specialized middle ear that collects airborne sounds through a tympanic membrane and delivers the vibrations to the inner ear via thin cartilaginous and ossified structures, usually involving the stapes [2]. Contrary to the classical view, the presence of a tympanic ear is no longer considered to be the ancestral condition for land vertebrates, and many of the so-called “otic notches“ in the basalmost, still aquatic tetrapods are now interpreted as spiracular notches, rather than as having hosted a tympanum [3]–[5]. Among anamniotic tetrapods, there is some indication that a tympanic ear had evolved in temnospondyls, and potentially in seymouriamorphs and diadectomorphs, whereas there is no evidence of its existence in basal amniotes [6]–[8]. A structural requirement for the stapes to serve as a hearing device is the loss of its function as a brace of the braincase against the dermal skull [7], [9]. In all basal members of the 3 major clades of Amniota (Synapsida, Eureptilia, and Parareptilia) the stapes is massive and strut-like, while the paroccipital process of the braincase is short and does not make contact with the skull roof. These same features are also found in the parareptilian millerettids, indicating that they lacked a tympanic ear as well, despite previous suggestion of the contrary [10]. Within amniotes, true tympanic ears were not thought to have evolved until the Mesozoic [7], [9], when they finally appear independently in basal mammals, lepidosaurs, archosaurs, and turtles. However, as evidence continues to mount supporting the (eureptilian) diapsid affinity of turtles [11]–[13], true tympanic ears matching all the above criteria have thus far been found in synapsids and eureptiles, but not in parareptiles.

The Middle Permian (Late Kazanian to Early Tatarian) Mezen River Basin, Central Russia, has yielded a variety of small terrestrial parareptiles (*Macroleter*, *Tokosaurus*, *Emeroleter*, *Bashkyroleter*, *Nycteroleter*, *Nyctiphruretus* [14]–[16]), one of which, *Macroleter*, has also been found in the Permian of the United States [17]. Recent detailed study of *Macroleter* has suggested a close relationship to the large pareiasaurs [18]; however, neither a comprehensive phylogenetic treatment considering all the relevant taxa, nor a functional interpretation of the cranial anatomy has been performed to date. Here, we report on the first phylogenetic study of all the relevant non-pareiasaurian parareptiles from the Mezen River Basin, and provide phylogenetic and anatomical evidence that these taxa include the first amniotes in which a highly specialized impedance-matching middle ear had evolved, suggesting ecological adaptations very different from those of contemporaneous tetrapods. The presence of such auditory specializations in not only one, but several species of parareptiles emphasizes the strong adaptive significance of these features and indicates that the ecological diversification of amniotes was already considerably advanced by the late Paleozoic.

## Results

### Phylogenetic Analysis

**Figure 1:**
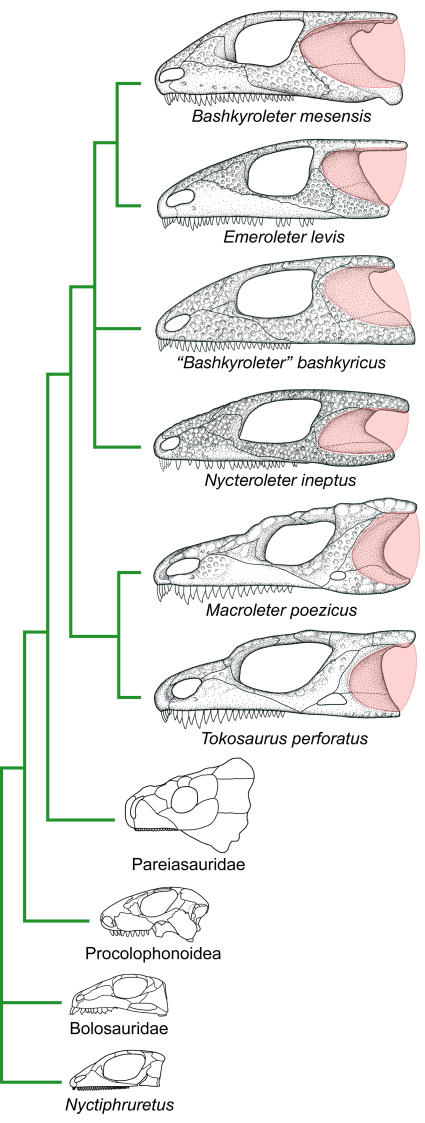
Phylogeny of the non-pareiasaurian parareptiles from the Mezen River Basin. With the exception of *Nyctiphruretus*, all relevant taxa form a clade that is sister to Pareiasauridae, and are characterized by the presence of a large temporal emargination indicative of a prominent tympanum (in pink). Not to scale.

In our largely revised and expanded analysis of parareptilian relationships [for details see Materials and Methods and the Supporting Information, Figure S1, Text S1, S2, Table S1], we obtained 6 equally parsimonious trees (tree length: 427; consistency index: 0.4848; homoplasy index: 0.5972; retention index: 0.6970; rescaled consistency index: 0.3379), with lack of resolution affecting only the positions of *Eudibamus/Belebey* and *Nyctiphruretus* relative to the remaining derived parareptiles, as well as the relationships between *“Bashkyroleter” bashkyricus*, *Nycteroleter*, and *Bashkyroleter mesensis/Emeroleter* (Figure 1). Except for *Nyctiphruretus*, all non-pareiasaurian parareptiles from the Mezen River Basin formed a monophyletic group, here informally termed the ‘nycteroleters,’ that was sister to Pareiasauridae within a larger clade that also included Procolophonoidea–the only known parareptiles to have survived the Permo-Triassic extinction event [19].

### Morphology of the temporal region and the braincase

**Figure 2:**
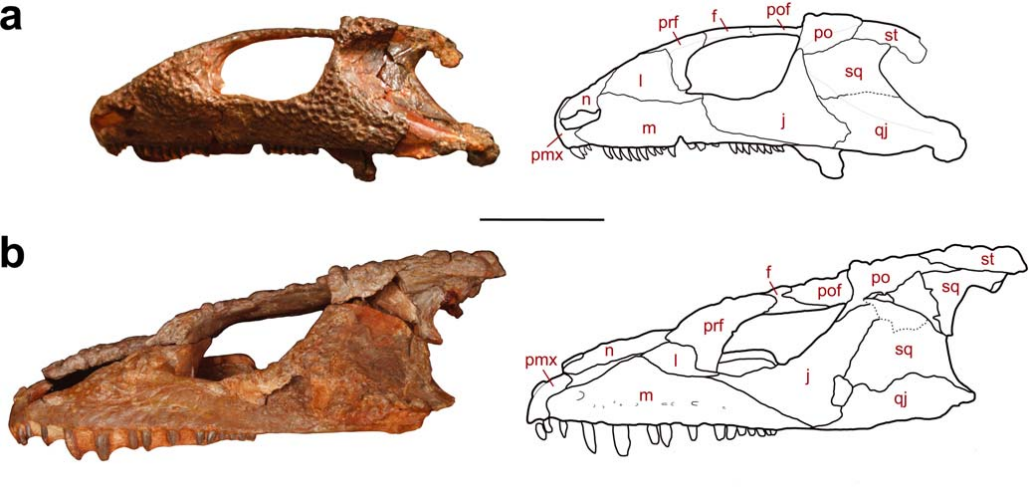
Hearing adaptations in the temporal region of the non-pareiasaurian parareptiles from the Mezen River Basin. (a) Lateral view of the skull of *Bashkyroleter mesensis* (PIN 162/30), which possesses the largest tympanum among the investigated taxa, as indicated by the non-sculptured posterolateral depression and the temporal emargination. (b) Lateral view of the skull of *Macroleter poezicus* (PIN uncataloged), the basalmost taxon within the clade. Bone abbreviations: f, frontal; j, jugal; m, maxilla; n, nasal; pmx, premaxilla; po, postorbital; pof, postfrontal; prf, prefrontal; qj, quadratojugal; sq, squamosal; st, supratemporal. Scale bar equals 2 cm.

A characteristic feature shared by *Macroleter*, *Tokosaurus*, *Emeroleter*, *Bashkyroleter*, and *Nycteroleter* is the presence of a distinct emargination at the posterolateral edge of the skull and a concave, smooth depression in the temporal region extending across most of the squamosal and large parts of the quadratojugal (Figure 1, Figure 2). The remaining area of the temporal region is characterized by distinctive dermal sculpturing, and a well developed, laterally protruding rim at its dorsal margin formed by the overhanging supratemporal and postorbital. The overall configuration is highly indicative of a large tympanum spanning the posterior emargination of the cheek, its dorsal edge, and the unsculptured depression, the latter feature implying that the skin was not closely applied to the bone. A tympanum of such a large relative size is not known in any other amniote and is among the largest ever recorded for tetrapods. The basal taxa *Macroleter* and *Tokosaurus* possess a small lower temporal fenestra and a tympanum restricted to the posterior half of the cheek. This fenestra is not present in the remaining species, a condition likely related to the dramatic anterior extent of the tympanum as indicated by the narrowly sculptured postorbital rim in the more derived taxa.

**Figure 3:**
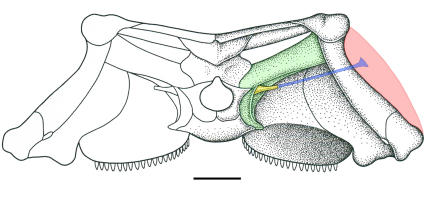
Middle ear reconstruction of *Macroleter poezicus* in occipital view. The paroccipital process (in green) is firmly attached to the dermal skull, the stapes (in yellow) is short and slender and was presumably connected to the tympanum (in pink) by an unossified extrastapes (in blue). Scale bar equals 1 cm.

**Figure 4:**
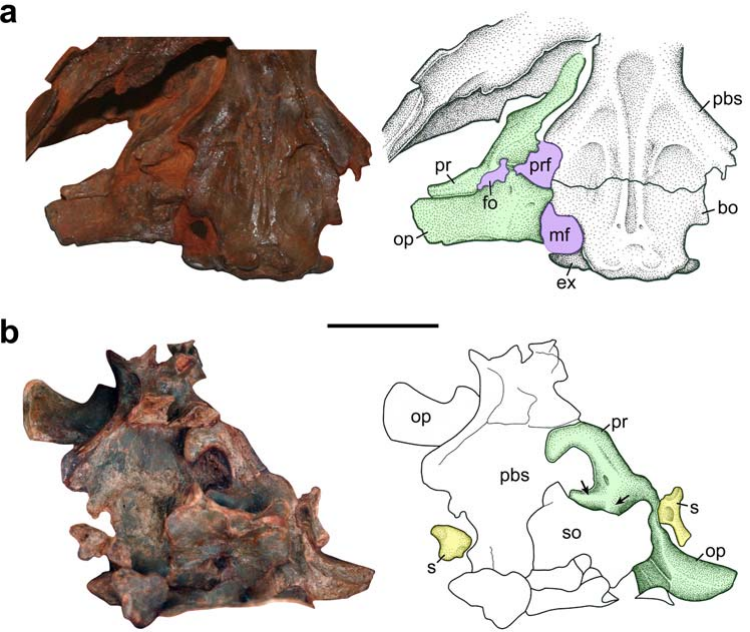
Hearing adaptations in the braincase of *Macroleter poezicus*. (a) Right half of braincase in ventral view (PIN 4609/1), showing the fenestra ovalis (fo), the pressure relief window (prf) and the metotic fissure (mf). Bone abbreviations: bo, basioccipital; pbs, parabasisphenoid; ex, exoccipital; op, opisthoticum; pr, prootic. (b) Disarticulated braincase in dorsal view (PIN uncataloged), showing the footplate (left) and the distal process (right) of the stapes (s), and the swellings on the inner side of the otic capsule (marked by arrows) indicating an ossified medial wall of the inner ear. Additional abbreviations: so, supraoccipital. Scale bar equals 1 cm.

The braincase is not completely preserved in each taxon, but is nicely documented in several specimens of *Macroleter*. The paroccipital processes are well developed and meet the dermal skull at its posterolateral corner (Figure 3). Unlike any contemporaneous amniote, the lateral wall of the braincase is subdivided into several distinct openings (Figure 4). The ancestrally large fenestra vestibuli is restricted to a small fenestra ovalis formed only by the opisthotic and prootic, which is also the case in modern amniotes possessing a sophisticated auditory apparatus [7]. Consequently, the large opening ventral to the fenestra is here interpreted as a pressure relief window to accommodate the vibrations of the inner ear fluid. Because the lateral wall of the braincase is largely unossified in basal amniotes, it remains unclear if this window is derived from a subdivision of the ancestral fenestra vestibuli or from a partition of the metotic fissure. The reinvestigation of another specimen of *Macroleter*, in which the anterior part of the internal area of the braincase is exposed, revealed several distinct swellings in the central section of the braincase wall, indicating that the medial wall of the otic capsule was ossified in a manner similar to that of modern amniotes with an impedance-matching middle ear (Figure 4). Additionally, both stapes are preserved in the same specimen, the left exposing the small footplate and the right showing an extremely narrow and short distal process (Figure 4). In all extant reptiles with a well-developed tympanic ear the stapes is slender, comparatively short, and does not reach the tympanic membrane, instead being connected to a cartilaginous extrastapes that mediates between the stapes and the tympanum [2]. The same configuration appears to be present in *Macroleter* and the other ‘nycteroleters.’ In addition there is a small opening close to the footplate of the right stapes that likely represents a pneumatic foramen analogous to those found in the stapes of owls [20].

## Discussion

### Functional comparison with modern amniotes

The configuration of the tympanic ear in these parareptiles is unique for amniotes in that it is not the quadrate as in other reptiles, or the tympanic (angular) as in mammals, but the squamosal and the quadratojugal to which the main parts of the tympanum are connected. In fact, this particular condition most closely resembles those described for anamniotes such as temnospondyls and seymouriamorphs [6], [7]. In order to test the effectiveness of impedance matching in the studied taxa, we calculated the ratio of tympanum area relative to the size of the stapedial footplate for *Macroleter*, noting that this is the basalmost taxon with the relatively smallest tympanum. Considering the entire tympanic area, a value of 35∶1 was determined, although since only two thirds of an eardrum are acoustically efficient [21] the corrected ratio is 23∶1; both values lie well within what is found in extant amniotes (13–42 in squamates [21], [22]; 11–47 in archosaurs [birds] [2], [20]; 8.5 in the slider turtle [23]; 19–46 in marsupials [24]; 14–60 in placentals [2]) and are likely to be higher in the remaining taxa of interest due to their larger tympanum. For a final approximation of the efficiency of impedance matching, the area ratio needs to be combined with the lever arm ratio, which considers the arrangement of either the ossicular chain or the cartilaginous extrastapes [2]; unfortunately, the latter is not preserved in the investigated taxa. Nonetheless, the area to footplate ratio suggests that the hearing abilities of *Macroleter* were at least comparable with those of an average lizard.

### Paleobiological implications of impedance matching

**Figure 5:**
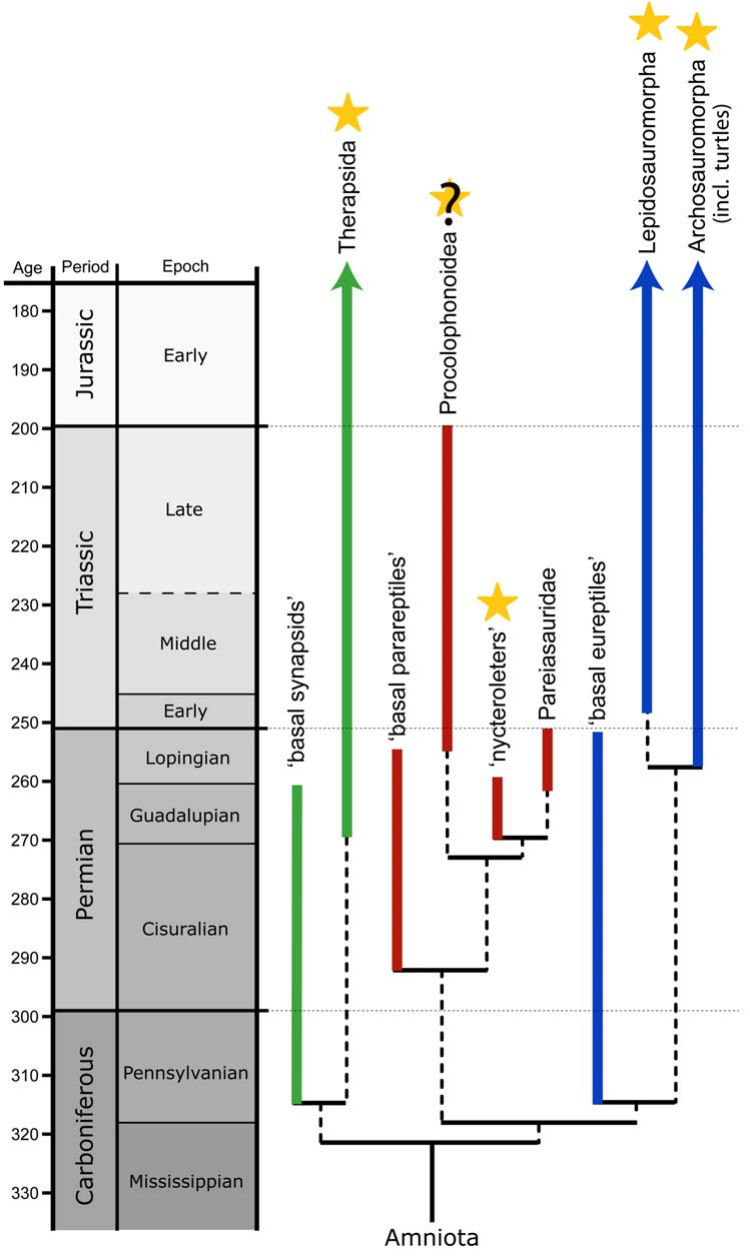
The occurrence of impedance-matching hearing within amniotes. A middle ear exhibiting the functional criteria for impedance-matching has thus far been recorded in synapsids, lepidosauromorphs, and archosauromorphs (marked with an asterisk), and was assumed not to have evolved before the Mesozoic; the presence of a tympanic middle ear in the taxa of our study (here informally named “nycteroleters”) represents the only known occurrence of such a structure in Paleozoic amniotes. Whether or not a true tympanic middle ear was also present in a closely related clade, the Procolophonoidea, remains unresolved.

Our study documents the first evidence of a true tympanic middle ear in Paleozoic amniotes (Figure 5), an evolutionary novelty that was hitherto believed to have evolved first in the Mesozoic [7], [9]. The evolution of an impedance-matching middle ear within Amniota has been interpreted to have occurred in concert with the diversification of modern insects, which reached its peak in the Mesozoic, implying that the buzzing sound of flying insects would have favoured the evolution of an advanced hearing sense [9], [25]. Considering that in modern amniotes the explicit use of a sophisticated auditory system, be it for prey capture or intraspecific communication, is most often found in crepuscular, nocturnal, and burrowing animals such as owls, cats, geckos or moles and mole-rats [20], [22], [26]–[30], an alternative explanation, at least with respect to the present study, might be that living in dim-light environments had become of selective advantage by the Middle Permian. This was potentially due to the increasing diversification of amniotes in the late Paleozoic, which would have resulted in a reduction of previously available niche space. Markedly enlarged orbits present in all of our investigated taxa also suggest non-diurnal life habits. In this context, future analyses might reveal that similar ecologies existed in the closely related procolophonoids, which also possess enlarged orbits and for whom, at least in part, a burrowing lifestyle has been proposed [31]; in fact, the potential presence of an impedance-matching middle ear was suggested for the Triassic taxon *Procolophon* [32], though the otic region of this taxon appears plesiomorphic and not as specialized as the taxa of our study. It is not unlikely, however, that future analyses will reveal that an advanced hearing sense was characteristic of the clade comprising Pareiasauridae, Procolophonoidea, and the taxa of our study, and became secondarily modified in the large pareiasaurs. If so, then adaptations to dim-light environments might have been the driving force not only in the diversification of the herein investigated taxa, but even more so in the procolophonoids, and were possibly an important factor in their survival of the Permo-Triassic extinction event. This interpretation can also be applied to Archosauriformes, another amniote clade that was not significantly affected by the Permio-Triassic extinction, and for which there is molecular data indicating that the clade Archosauria, which is nested within Archosauriformes, was ancestrally nocturnal [33]. In addition, the fact that Early Triassic synapsids living immediately after the extinction apparently had specific burrowing life habits [34] also suggests that adaptations to dim-light environments were potentially an important factor in the survival of the Permo-Triassic transition.

By the end of the Paleozoic many of the major adaptive features characterizing amniote evolution had evolved; important examples include the ability for flight, secondary aquatic lifestyle, and high-fiber herbivory [35]–[37]. The discovery of a highly-evolved auditory apparatus in Middle Permian parareptiles even further emphasizes that the entire groundplan for the impressive evolutionary history of amniotes was already largely in place by the end of the Paleozoic; what followed was in fact only a subsequent tinkering of earlier inventions.

## Materials and Methods

### Parareptilian specimens from the Mezen River Basin examined for this study

Institutional abbreviations: PIN–Palaeontological Institute of the Russian Academy of Sciences Moscow; UTM–University of Toronto at Mississauga, Canada.

*Bashkyroleter mesensis*: PIN 162/30, 3586/17, 3706/5, 3706/32, 4541/3, 4541/4, 4639/2, 4653/5, 4659/18.

*“Bashkyroleter” bashkyricus*: PIN 164/3, 164/60.

*Emeroleter levis*: PIN 2212/14, 2212/41, 2212/81, 2212/84, 2212/89, 2212/92.

*Macroleter poezicus*: PIN 3586/1, 3706/61, 4543/3, 4609/1 (originally labelled as *Nycteroleter*), PIN uncataloged immature specimen, UTM/Mezen/2001/1, UTM/Mezen/2001/2.

*Nycteroleter ineptus*: PIN 3717/27.

*Nyctiphruretus acudens*: PIN 158/4, 158/5, 158/6, 158/7, “162”, 162/1, 162/29, 3586/79, 4657/3, 4659/1, 4659/2, 4659/14, 4660/9, 4660/18, two uncataloged specimens.

*Tokosaurus perforatus*: PIN 104B/2004, 104B/2028.

### Phylogenetic analysis

The phylogenetic analysis of Parareptilia was performed using the software packages PAUP* 4.0 and MacClade [38], [39] using 137 parsimony-informative characters and 28 taxa. Both data matrix and character list represent largely revised and expanded versions of previous analyses of parareptilian relationships [18], [40], [41]. For the first time all of the non-pareiasaurian parareptiles from the Mezen River Basin of Russia were included in a single data set (*“Bashkyroleter” bashkyricus, Bashkyroleter mesensis, Emeroleter levis, Macroleter poezicus, Nycteroleter ineptus, Nyctiphruretus acudens, Tokosaurus perforatus*), with the exception of *Rhipaeosaurus tricuspidens*, which is only partially preserved and requires further investigation. *Seymouria*, Diadectidae, and Limnoscelidae were used as outgroup taxa. The heuristic search option (random stepwise addition, multistate taxa interpreted as polymorphism) was used for the final analysis [see the Supporting Information for the list of apomorphies (Text S1), character list (Text S2), data matrix (Table S1), and the consensus tree (Figure S1) of all the included taxa, along with the bootstrap values].

### Calculation of area ratio

The area ratio for *Macroleter* was determined with the imaging software ImageJ (http://rsb.info.nih.gov/ij), using digital photographs for measuring the stapedial footplate and a two-dimensional skull reconstruction (created in Adobe Photoshop CS 8.0.1 on the basis of digital measurements and drawings) for estimating the outline of the tympanum; in order to avoid overestimates of the tympanic extent a conservative approach was taken assuming a straight line between the posterodorsal and posteroventral edge of the skull.

## Supporting Information

Text S1 List of apomorphies for the major nodes within Parareptilia.
(0.04 MB PDF)

Text S2 List of characters used for the phylogenetic analysis of Parareptilia.
(0.14 MB PDF)

Table S1 Data matrix used for the phylogenetic analysis of Parareptilia.
(0.25 MB PDF)

Figure S1 Strict consensus tree of the phylogenetic analysis with bootstrap values (1000 replicates.)
(0.01 MB PDF)
