# Supplementary material for: Impedance-Matching Hearing in Paleozoic Reptiles: Evidence of Advanced Sensory Perception at an Early Stage of Amniote Evolution
Source: PLoS One. 2007 Sep 12;2(9):e889. doi: 10.1371/journal.pone.0000889 (PMC1964539; doi:10.1371/journal.pone.0000889)
Supplement: Text S1 — List of apomorphies for the major nodes within Parareptilia. (0.04 MB PDF) [file pone.0000889.s001.pdf]

List of apomorphies for the major nodes within Parareptilia:

Parareptilia:

Unequivocal: #10(1), #23(1), #32(1), #40(1), #104(1), #131(1).

ACCTRAN: #11(2), #24(0), #51(1), #77(2), #91(1), #99(1), #112(1), #117(1).

DELTRAN: #120(1).

Ankyramorpha:

Unequivocal: #5(1), #18(1), #30(1), #45(1), #58(1), #72(1), #80(1), #88(1), #100(1), #101(1).

ACCTRAN: #29(1), #63(1), #79(1), #83(1), #93(0), #96(2).

DELTRAN: #31(1), #57(1).

“Nycteroleters”:

Unequivocal: #36(1), #58(2), #81(1), #84(0).

ACCTRAN: #6(1), #12(0), #23(0), #46(0), #48(0), #73(2), #93(1).

DELTRAN: #134(0). (Remark: this character refers to the temporal emargination which we interpret to have hosted a tympanum; however, the apomorphy did not turn out to be unequivocal because Procolophonoidea also possess an emargination, emphasizing the need for more information about the functional anatomy of derived parareptiles [see also the discussion in the text]).
