# Supplementary material for: Impedance-Matching Hearing in Paleozoic Reptiles: Evidence of Advanced Sensory Perception at an Early Stage of Amniote Evolution
Source: PLoS One. 2007 Sep 12;2(9):e889. doi: 10.1371/journal.pone.0000889 (PMC1964539; doi:10.1371/journal.pone.0000889)
Supplement: Text S2 — List of characters used for the phylogenetic analysis of Parareptilia. (0.14 MB PDF) [file pone.0000889.s002.pdf]

List of characters used for the phylogenetic analysis of Parareptilia. Characters marked with an asterisk are new to the analysis of parareptilian ingroup relationships. Cited references can be found at the end of the list.

1. Narial shelf: absent (0); present (1). (Laurin and Reisz 1995 #1)
2. Frontal orbital contact: absent (0); present (1). (Laurin and Reisz 1995 #2)
3. Frontal lateral lappet: absent (0); large, occupies at least one-third of the dorsal margin of the orbit (1). (deBraga and Reisz 1996 #7)
4. Pineal foramen position: in the middle of the body of the parietal (0); displaced posteriorly (1); displaced anteriorly and reaches level of orbit (2); absent (3). (Modified from deBraga and Rieppel 1997 #49)
5. Postparietal: paired (0); median (1); greatly reduced or absent (2). (Modified from Laurin and Reisz 1995 #4)
6. Postparietal position: dorsally exposed, integrated into skull table (0); occipital (1). (Laurin and Reisz 1995 #5)
7. Prefrontal-palatal contact: absent (0); weak (1); strong (2). (Laurin and Reisz 1995 #6)
8. Prefrontal medial flange: narrow (0); wide (1). (Laurin and Reisz 1995 #7)
9. Bulbous medial process of prefrontal: absent (0); present (1). (Laurin and Reisz 1995 #8)
10. Lacrimal narial contact: present (0); absent (1). (Laurin and Reisz 1995 #9)
11. Foramen orbitonasale: absent (0); represented by a medial indentation on the lacrimal and a dorsal indentation on the palatine (1); enclosed between prefrontal, lacrimal and palatine (2). (Laurin and Reisz 1995 #10)
12. Jugal anterior process: does not extend to anterior orbital rim (0); extends at least to level of anterior orbital rim (1). (Laurin and Reisz 1995 #11)
13. Postorbital posterior process shape: slender, half as wide as it is long (0); increased width, parallelogram outline in lateral aspect (1). (deBraga and Reisz 1996 #14)

14. Squamosal-parietal contact: present (0); absent (1). (Modified from Laurin and Reisz 1995 #12)
15. Posterolateral corner of skull roof; formed by tabular (0); formed mostly by supratemporal (1); formed by parietal and small supratemporal or parietal alone (2). (Laurin and Reisz 1995 #15)
16. Tabular size: large and part of skull table (0); small and largely occipital (1); absent (2). (Laurin and Reisz 1995 #17)
17. Supratemporal size: large (0); small (1); absent (2). (Laurin and Reisz 1995 #18)
18. Premaxillary dorsal process: broad, narial opening faces predominantly laterally (0); narrow, narial opening faces anteriorly (1). (deBraga and Reisz 1996 #1)
19. Anterodorsal process of the maxilla: absent (0); present (1). (Modified from Laurin and Reisz 1995 #19)
20. Anterior lateral maxillary foramen: equal in size to other maxillary foramina (0); larger than other foramina (1); the lateral surface of the maxilla lacks large foramina (2). (Laurin and Reisz 1995 #20)
21. Maxilla and quadratojugal: in contact (0); separated (1). (Laurin and Reisz 1995 #22)
22. Quadratojugal anterior extent: reaches posterior border of orbit (0); does not reach level of posterior border of orbit (1). (Modified from Laurin and Reisz 1995 #23)
23. Caniniform region: present (0); absent (1). (Laurin and Reisz 1995 #24)
24. Single caniniform maxillary tooth: absent (0); present (1). (Laurin and Reisz 1995 #25)
25. Squamosal and post-temporal fenestra: separated (0); in contact (1). (Laurin and Reisz 1995 #26)
26. Quadratojugal shape: does not reach beyond the level of the ventral orbital margin (0); extends dorsally beyond the level of ventral orbital margin (1). (Modified from Laurin and Reisz 1995 #28)

27. Quadratojugal ornamentation: confluent with the cheek and not ornate in any manner (0); ornamented, dermal protuberances project from its surface (1). (deBraga and Rieppel 1997 #43)
28. Upper temporal fenestra: absent (0); present (1). (Laurin and Reisz 1995 #29)
29. Ventral temporal emargination: absent (0); present and bounded ventrally, forming a lower temporal fenestra (1); present and open ventrally (2). (Modified from Laurin and Reisz 1995 #30)
30. Postorbital contribution to lateral temporal fenestra: bordered by jugal, quadratojugal, squamosal, postorbital (0); no contribution by postorbital (1). (deBraga and Reisz 1996 #20)
31. Quadratojugal-lateral temporal fenestra contribution: quadratojugal excluded from posterior border (0); quadratojugal contributes to lateral temporal fenestra (1). (deBraga and Reisz 1996 #16)
32. Postorbital region of skull: length at least equals anteroposterior extension of orbit (0); postorbital region shorter than anteroposterior extension of orbit (1). (Modified from Laurin and Reisz 1995 #32)
33. Ventral margin of postorbital skull region: expanded below ventral extent of maxilla (0); rectilinear (1); emarginated (2). (Modified from Laurin and Reisz 1995 #33)
34. Quadrate lateral exposure: absent (0); present (1). (Laurin and Reisz 1995 #34)
35. Quadrate anterior process: long (0); short (1). (Laurin and Reisz 1995 #35)
36. Jaw articulation position: posterior to occiput (0); even with occiput (1); anterior to occiput (2). (Laurin and Reisz 1995 #36)
37. Posterior extension of orbit: absent (0); present (1). (Laurin and Reisz 1995 #37)
38. Dermal sculpturing: absent (0); tuberosities (1); tuberosities and pits (2); honeycomb pattern of ridges and pits (3). (Laurin and Reisz 1995 #38)

39. Sculpturing involving circumorbital bumps: no distinctive ornamentation (0); circumorbital tubercles (1). (Tsuji 2006 #45)
40. Posterior margin of skull roof: roughly straight (0); with a single, median embayment (1); embayed bilaterally (2). (Modesto 1999 #125)
41. Interpterygoid vacuity anterior extent: absent (0): reaches beyond posterior border of palatine (0); reaches level of palatine or less (1). (Reisz et al. 2007)
42. Choana: parallel to maxilla; palatine forms its posterior edge only (0); curved posteromedially; palatine forms its posterior and part of its lateral edge (1). (Laurin and Reisz 1995 #40)
43. Alar flange of the vomer: absent (0); present (1). (Tsuji 2006 #50)
44. Arcuate flange of pterygoid: present (0); absent (1). (Laurin and Reisz 1995 #42)
45. Cranio-quadrate space: small, quadrate ramus of pterygoid and paraoccipital process of opisthotic converge posterolaterally (0); large, quadrate ramus of pterygoid and paraoccipital process of opisthotic are parallel to each other (1). (Laurin and Reisz 1995 #43)
46. Pterygoid anterior extent: reaches level of posterior end of choana (0); posterior to choana (1). (Laurin and Reisz 1995 #44)
47. Transverse flange of the pterygoid: large, approaches cheek, a noticeable lateral projection (0); small, does not approach cheek (1). (Modified from Lee 1997 #19)
48. Transverse flange of pterygoid orientation: directed posterolaterally or transversely (0); directed anterolaterally (1); directed anteriorly (2). (Modified from Laurin and Reisz 1995 #45)
49. Transverse flange of pterygoid dentition: shagreen of denticles, no ventral ridge (0); single row of large teeth, no ventral ridge (1); edentulous with ventral ridge (2). (Laurin and Reisz 1995 #46)

50. Quadrate ramus of pterygoid: merges smoothly into transverse flange without distinctive excavation (0); deep excavation on posterolateral surface (1). (deBraga and Reisz 1996 #29)
51. Ectopterygoid dentition: present (0); absent (1). (Laurin and Reisz 1995 #48)
52. Ectopterygoid relationship to transverse flange: ectopterygoid distal to transverse flange, does not contribute to flange (0); ectopterygoid makes contact with transverse flange (1). (deBraga and Reisz 1996 #33)
53. Suborbital foramen: absent (0); present (1); fenestra present (2). (Laurin and Reisz 1995 #49)
54. Basicranial articulation: kinetic/synovial (0); sutured and/or immobile (1). (Modified from Lee 1997 #2)
55. Length of basicranial articulation: restricted to anterolateral margin of the parasphenoid (0); extends over much of length of main body of parasphenoid (1). (deBraga and Reisz 1996 #36)
56. Parasphenoid pocket for cervical musculature: present (0); absent (1). (Laurin and Reisz 1995 #50)
57. Parasphenoid wings; present, parasphenoid broader posteriorly than long (0); absent, parasphenoid narrower posteriorly than long (1). (Modified Laurin and Reisz 1995 #51)
58. Cultriform process: longer than the body of the parasphenoid (0); shorter than the body of the parasphenoid (1); absent (2). (Laurin and Reisz 1995 #52)
59. Parasphenoid teeth: absent (0); present (1). (Laurin and Reisz 1995 #53)
60. Supraoccipital: absent (0); plate-like, no saggital crest (1); body constricted at midline, forming saggital crest (2). (Modified from deBraga and Rieppel 1997 #56)
61. Paroccipital process: vertically broad (0); antero-posteriorly expanded (1); narrow (2); tubular, composed of opisthotic (3). (Laurin and Reisz 1995 #56)

62. Paraoccipital process orientation: directed primarily laterally (0); oriented obliquely, at an angle of at least 45 degrees from the horizontal plane of the skull (1). (deBraga and Reisz 1996 #44)
63. Sutural contact between paroccipital process and dermatocranium: absent (0); present (1). (Modified Laurin and Reisz 1995 #57)
64. Otic trough in ventral flange of opisthotic: absent (0); present (1). (Laurin and Reisz 1995 #58)
65. Medial wall of inner ear (made of prootic): unossified (0); ossified with acoustic nerve foramina (1). (Laurin and Reisz 1995 #59)
66. Post-temporal fenestra: absent (0); small, diameter less than half the diameter of foramen magnum (1); large, diameter at least equal to foramen magnum (2). (deBraga and Rieppel 1997 #59)
67. Osseous contact between basioccipital and basisphenoid: present (0); absent (1). (Lee 1993 #A3, scored as per Laurin and Reisz 1995 #61)
68. Occipital condyle shape: transversely broad (0); reniform to circular (1). (Laurin and Reisz 1995 #62)
69. Ventral exposure of basioccipital: contributes extensively to ventral surface of the braincase (0); restricted to condylar region (1). (deBraga and Reisz 1996 #37)
70. Ventral braincase tubera: absent (0); present and restricted to basioccipital (1); present, very large and restricted to basisphenoid (2); median (3). (Modified from Laurin and Reisz 1995 #63, and deBraga and Rieppel 1996 #65)
71. Lateral flange of exoccipital: absent (0); present (1). (Laurin and Reisz 1995 #64)
72. Quadrate condyle articular surfaces: strongly convex, antero-posteriorly longer than they are wide (0); nearly flat, antero-posteriorly shorter than they are wide (1). (Modified from Laurin and Reisz 1995 #65)

73. Stapes: robust, greatest depth exceeding one-third of total length (0); slender, length at least four times depth (1); slender but short (2). (Modified from deBraga and Rieppel 1997 #45)
74. Stapedial dorsal process: ossified (0); unossified (1). (Laurin and Reisz 1995 #67)
75. Labyrinthodont infolding: present (0); absent (1). (Laurin and Reisz 1995 #68)
76. Morphology of marginal dentition: single cusp (0); two to seven cusps (1); more than seven cusps (2). (Modified from Lee 1997 #59)
77. Foramen intermandibularis: anterior symphysial foramen (0); two foramina, a symphysial and a posterior foramen located anterior to coronoid process (1); two foramina, a symphysial and a posterior foramen located located posterior to or at level of coronoid process (2). (Laurin and Reisz 1995 #69)
78. Meckelian fossa orientation: faces mediodorsally, prearticular narrow (0) faces dorsally, prearticular broad (1). (Laurin and Reisz 1995 #70)
79. Fossal meckelii: long, occupies at least 20% of lower jaw length (0); short, occupies less than 20% of lower jaw length (1). (Laurin and Reisz 1995 #71)
80. Surangular length: extends beyond coronoid eminence (0); does not extend beyond coronoid eminence (1). (Laurin and Reisz 1995 #72)
81. Accessory lateral shelf on surangular anterior to articular region: absent (0); present (1). (Laurin and Reisz 1995 #73)
82. Coronoid number: two or three (0); one (1). (Laurin and Reisz 1995 #74)
83. Prearticular extends: beyond the coronoid eminence (0); does not extend beyond coronoid eminence (1). (Modified from Laurin and Reisz 1995 #75)
84. Retroarticular process: absent or small and narrow (0); transversely broad, dorsally concave (1). (Laurin and Reisz 1995 #76)
85. Retroarticular process composition: articular body (0); three or more elements (articular, prearticular, angular and surangular) (1). (Laurin and Reisz 1995 #77)

86. Lateral shelf on articular region: absent (0); on articular (1); on surangular (2). (Laurin and Reisz 1995 #78)

87. Coronoid process: small or absent, composed of several elements (0); high process composed of coronoid only (1); high, composed primarily of dentary (2). (Modified from Laurin and Reisz 1995 #79)

88. Splenial: contributes to symphysis (0); excluded from symphysis (1). (Laurin and Reisz 1995 #80)

89. Presacral vertebral count: more than twenty (0); twenty or less (1). (Laurin and Reisz 1995 #81)

90. Axial centrum orientation: in plane of axial skeleton (0); sloping anterodorsally (1). (Laurin and Reisz 1995 #82)

91. Atlantal neural arch: possesses epipophysis (0); lacks epipophysis (1). (Lee 1995, scored as per Modesto 1999 #126)

92. Axial intercentrum: with rounded anteroventral edge (0); with strong anterior process (1). (Laurin and Reisz 1995 #84)

93. Atlantal pleurocentrum and axial intercentrum: separate elements (0); attached or fused (1). (Laurin and Reisz 1995 #85)

94. Trunk neural arches: swollen (0); narrow (1). (Modified from Laurin and Reisz 1995 #86)

95. Ventral surface of anterior pleurocentra: ventral surface of vertebral centra uniform (0); ventral surface of vertebral centra bearing an exavation on either side of the midline, coupled with a flattened median crest between them (1). (Modified from Laurin and Reisz 1995 #87)

96. Number of sacral vertebrae: one (0); two (1); three or four (2). (Laurin and Reisz 1995 #88)

97. Sacral rib distal overlap: broad with narrow gap between ribs (0); small or absent with wide gap between ribs (1). (Laurin and Reisz 1995 #89)

98. Transverse process or ribs: present only on a few anterior caudals (0); present on at least thirteen caudals (1). (Laurin and Reisz 1995 #90)

99. Caudal hemal arches: wedged between centra (0); attached to anterior centrum (1). (Laurin and Reisz 1995 #91)

100. Interclavicle: diamond-shaped (0); T-shaped, with long, slender lateral processes (1). (Laurin and Reisz 1995 #92)

101. Interclavicle attachment for clavicle: ventral sutural area (0); anteriorly directed groove (1); tightly sutured into plastron (2). (Laurin and Reisz 1995 #93)

102. Cleithrum: caps scapula anterodorsally (0); does not cap scapula at all (1); absent (2). (Laurin and Reisz 1995 #94)

103. Scapula: broad (0); narrow, thin (1). (Laurin and Reisz 1995 #96)

104. Supraglenoid foramen: present (0); absent (1). (Laurin and Reisz 1995 #97)

105. Glenoid: anteroposteriorly long, helical (0); short, bipartite (1). (Laurin and Reisz 1995 #98)

106. Acromion: absent (0); present (1). (Laurin and Reisz 1995 #99)

107. Sternum: not mineralized (0); mineralized (1). (Laurin and Reisz 1995 #100)

108. Supinator process: strongly angled relative to shaft, separated from it by groove (0); parallel to shaft, separated from it by groove (1); parallel to shaft, not separated from shaft (2). (Laurin and Reisz 1995 #101)

109. Ectepicondylar foramen: only groove present (0); groove and foramen present (1); only foramen present (2); both absent (3). (Laurin and Reisz 1995 #102)

110. Entepicondylar foramen: present (0); absent or not fully enclosed (1). (Laurin and Reisz 1995 #103)

111. Humerus: with robust heads and a short shaft (0); short and robust, without a distinct shaft (1); slender with long shaft (2). (Laurin and Reisz 1995 #104)

112. Olecranon process: large, proximal articular facet of ulna faces medially (0); small or absent (1). (Modified from Laurin and Reisz 1995 #105)

113. Manual phalangeal formula: 2 3 4 5 3 (0); 2 3 4 4 3 (1); 2 3 3 3 3 or less (2). (Laurin and Reisz 1995 #106)

114. Dorsolateral shelf on iliac blade: absent (0); present (1). (Laurin and Reisz 1995 #107)

115. Iliac blade: low, with long posterior process (0); dorsally expanded, distally flaring (1). (Laurin and Reisz 1995 #108)

116. Acetabular buttress: small, overhangs acetabulum only moderately (0); large, overhangs acetabulum strongly (1). (Laurin and Reisz 1995 #109)

117. Oblique ventral ridge of femur (adductor crest): present (0); absent (1). (Laurin and Reisz 1995 #110)

118. Femoral proximal articulation: antero-posteriorly long (0); round (1). (Laurin and Reisz 1995 #111)

119. Greater trochanter of femur; absent (0); present on posterior edge of femur (1). (Laurin and Reisz 1995 #112)

120. Femoral shaft: short and broad (0); long and slender (1). (Laurin and Reisz 1995 #113)

121. Carpus and tarsus: short and broad (0); long and slender (1). (Laurin and Reisz 1995 #114)

122. Astragalus: absent (0); incorporates incompletely fused tibiale, intermedium, and perhaps centrale 4 (1); without traces of compound origin (2). (Laurin and Reisz 1995 #115)

123. Tibio-astragalar joint: flat (0); tibial ridge fits into astragalar groove (1). (Laurin and Reisz 1995 #116)

124. Astragalus and calcaneum: separate (0); sutured or fused (1). (Laurin and Reisz 1995 #117)

125. Medial pedal centrale: present (0); absent (1). (Laurin and Reisz 1995 #118)

126. Number of distal tarsals: five (0); four or less (1). (Laurin and Reisz 1995 #119)

127. Metapodials: not overlapping (0); overlapping (1). (Laurin and Reisz, 1995 #121)

128. Pedal phalangeal formula: 2 3 4 5 4 or 3 (0); 2 3 4 4 3 (1); 2 3 3 4 3 or less (2). (Laurin and Reisz 1995 #122)

129. Ratio between length of metatarsal one to length of metatarsal four: at least 0.5 (0); less than 0.5 (1). (Laurin and Reisz 1995 #123)

130. Dorsal dermal ossifications: absent (0); present (1). (Laurin and Reisz, 1995 #124)

131. Subtemporal process of jugal: present (0): absent (1). *

132. Dorsal extent of jugal: does not reach beyond the mid-level of the orbit (0); reaches the dorsal level of the orbit (1). *

133. Suture between jugal and maxilla: straight, jugal thins out smoothly towards anterior direction (0); “stepped”, anterior most tip of jugal very narrow but expands broadly posteriorly along with a dramatic thinning of the posterior process of the maxilla (1). *

134. Temporal notch: present (0); absent (1). *

135. Temporal depression associated with posterolateral excavation: restricted to the posterior half of the cheek (0); closely approaches the orbital margin (1). *

136. Contact between maxilla and prefrontal: absent (0); present (1). *

137. Contribution of maxilla to external naris: maxilla is either excluded from naris or forms only its ventral/posterior edge (0); maxilla extends also to the posterodorsal margin of naris (1). *

References

deBraga M, Reisz RR (1996) The early Permian reptile *Acleistorhinus pteroticus* and its phylogenetic position, J Vert Paleont 16: 384-395.

deBraga M, Rieppel O (1997) Reptile phylogeny and the interrelationships of turtes, Zool J Linn Soc, 120: 281-354.

Laurin M,Reisz RR (1995) A reevaluation of early amniote phylogeny, Zool J Linn Soc 113: 165-223.

Lee MSY (1993) The origin of the turtle body plan: bridging a famous morphological gap, Science 261: 1716-1720.

Lee MSY (1995) Historical burden in systematics and the interrelationships of 'parareptiles', Biol Rev 70: 459-547.

Lee MSY (1997) Pareiasaur phylogeny and the origin of turtles, Zool J Linn Soc 120: 197-280.

Modesto SP (1999) Observations on the structure of the early Permian reptile *Stereosternum tumidum* Cope, Palaeontologia Africana 35: 7-19.

Reisz RR, Müller J, Tsuji L, Scott D (2007) The cranial osteology of *Belebey vegrandis* (Parareptilia: Bolosauridae) from the Middle Permian of Russia, and its bearing on reptilian evolution, Zool J Linn Soc 151: 191-214.

Tsuji LA (2006) Cranial anatomy and phylogenetic affinities of the Permian parareptile *Macroleter poezicus*, J Vert Paleont. 26: 849-865.
