# Supplementary material for: Impedance-Matching Hearing in Paleozoic Reptiles: Evidence of Advanced Sensory Perception at an Early Stage of Amniote Evolution
Source: PLoS One. 2007 Sep 12;2(9):e889. doi: 10.1371/journal.pone.0000889 (PMC1964539; doi:10.1371/journal.pone.0000889)
Supplement: Table S1 — Data matrix used for the phylogenetic analysis of Parareptilia. (0.25 MB PDF) [file pone.0000889.s003.pdf]

Data matrix used for the phylogenetic analysis of Parareptilia.

	1									10										20
Seymouria	0	0	0	0	0	0	1	0	0	0	0	0&1	0	0	0	0	0	0	0	0
Limnoscelidae	0	0	0	0	1	1	0	0	0	0	0	1	1	1	1	0	0	0	0	0
Diadectidae	0	0	0	0	1	1	1	0	0	0	?	0	?	1	1	0	0	0	0	0
Synapsida	0&1	1	0	1&2	0	1	0	0	0	0	0	0	1	1	1	0	0	0	0	0
Mesosauridae	0	1	0	0	0	1	0	0	0	1	?	0	?	0	1	1	0	0	0	0
Eunotosaurus	?	1	0	2	0	1	?	?	?	?	?	0	0	1	1	1	0	?	1	?
Millerettidae	0	1	0	1	0	1	1	0	0	1	2	0	0	1	1	1	0	0	0	1
Eudibamus	0	1	0	?	?	?	2	1	0	?	?	0	1	?	2	?	2	1	0	?
Belebey	0	1	0	1	2	1	2	1	0	0	2	0	1	0	2	0	2	1	0	1
Acleistorhinus	0	1	1	0	1	1	?	?	?	1	?	0	1	1	1	1	0	1	1	1
Lanthanosuchus	0	1	1	0	2	1	1	0	0	1	?	0	1	1	1	1	0	1	1	?
Bradysaurus	0	0	0	0	1	0	1	0	0	0	?	1	1	1	1	2	0	1	1	1
Pareiasuchus	0	0	0	0	1	0	1	0	0	0	?	1	1	1	1	1	0	1	1	1
Scutosaurus	0	0	0	0	1	0	1	0	0	0	?	1	1	1	1	1	0	1	1	1
Procolophon	1	1	0	2	2	?	2	1	1	1	2	1	1	1	1	1	0	1	1	1
Owenetta	1	1	0	2	0	0	2	1	1	1	?	1	1	1	1	2	0	1	1	1
Barasaurus	1	1	0	2	0	0	?	1	1	0	?	0	1	1	1	2	0	1	1	1
Captorhinidae	0	1	0	0&2	0	1	1	0	0	0	1	1	1	0	2	2	1	0	0	0
Paleothyris	0	1	0	1	0	1	0	0	0	0	?	0	?	0	2	1	1	0	0	0
Araeoscelidia	0	1	0	0	0	1	0	0	0	0	0	0&1	0	0	2	1	1	?	0	0
Younginiformes	0	1	0	0	0	1	0	0	0	1	0	1	0	0	2	1	1	?	1	0
Macroleter	0	1	0	0	0&1	1	2	1	0	1	2	1	1	1	1	1	0	1	1	1
Bashkyroleter mesensis	0	1	0	0	1	1	2	1	0	1	2	0&1	1	1	1	1	0	1	1	1
"Bashkyroleter" bashkyricus	0	1	0	2	?	1	2	1	0	1	2	0	1	1	1	1	0	?	1	?
Nycteroleter	0	1	0	0	0	1	2	1	0	1	2	0	1	1	1	1	0	1	1	1
Emeroleter	0	1	0	0	?	?	2	1	0	1	2	0	1	1	1	?	0	1	1	1
Tokosaurus	0	1	0	2	?	1	2	1	0	1	?	0	1	1	1	?	0	1	1	1
Nyctiphruretus	0	1	0	1	1	1	2	1	0	1	2	0	1	1	1	1	0	1	1	1

	21									30										40
Seymouria	0	0	0	0	0	0	0	0	0	?	?	0	0	0	0	0	0	3	0	0
Limnoscelidae	0	1	0	0	0	1	0	0	0	?	?	0	0	0	0	1	0	0	0	1
Diadectidae	0	1	1	0	0	1	0	0	0	?	?	0	0	1	1	1	0	1	0	2
Synapsida	1	0&1	0	1	0	0	0	0	1	0	0	0	0&1	0	0	1	0	0	0	2
Mesosauridae	0	0	1	0	1	0	0	0	0	?	?	1	1	0	0	1	0	0	0	1
Eunotosaurus	1	1	1	0	1	1	0	0	0	?	?	1	2	1	0	2	0	1	0	1
Millerettidae	0	1	0&1	0	0	1	0	0	0&1	0	0	1	1	1	0	2	0	1	0	1
Eudibamus	?	0	1	0	1	?	0	0	?	1	1	1	?	0	0	0	?	0	0	0
Belebey	0	0	1	0	1	0	0	0	1	1	1	1	0	0	0	1	1	0	0	0
Acleistorhinus	0	1	0	1	1	1	0	0	1	1	1	0	1	0	0	2	0	1	0	1
Lanthanosuchus	1	1	1	0	1	1	1	0	1	1	1	0	1	0	0	0	0	2	0	1
Bradysaurus	1	0	1	0	0	0	1	0	0	?	?	0	0	0	0	2	0	2	1	1
Pareiasuchus	1	0	1	0	0	0	1	0	0	?	?	0	0	0	0	2	0	2	1	1
Scutosaurus	0	0	1	0	0	0	1	0	0	?	?	0	0	0	0	2	0	2	1	1
Procolophon	1	0	1	0	0	1	0	0	2	?	?	1	2	0	0	2	1	0	0	0
Owenetta	1	0	0	0	0	1	0	0	2	?	?	1	2	0	0	2	1	0	0	0
Barasaurus	1	0	1	0	0	1	0	0	2	?	?	1	2	0	0	2	1	0	0	1
Captorhinidae	0	1	0	1	1	0	0	0	0	?	?	0	1	0	1	1	0	3	0	2
Paleothyris	0	1	0	1	1	0	0	0	0	?	?	0	1	0	1	1	0	0	0	2
Araeoscelidia	0	0&1	0	1	0&1	0	0	1	1	0	1	0	1	0	1	1	0	0	0	2
Younginiformes	0	1	0	0	1	0	0	1	1	0	1	0	1	1	1	1	0	0	0	1
Macroleter	0	0	0	0	0	0	1	0	1	1	1	0	0	0	0	1	1	2	1	1
Bashkyroleter mesensis	1	1	0	0	0	1	1	0	0	?	?	0	1	0	0	?	1	2	0	1
"Bashkyroleter" bashkyricus	?	?	?	?	0	?	1	0	0	?	?	0	0	0	?	?	1	2	0	0
Nycteroleter	1	1	1	0	0	?	?	0	?	?	?	0	0	0	0	1	1	2	0	1
Emeroleter	0	1	0	0	?	?	1	0	0	?	?	0	1	?	0	1	1	2	0	1
Tokosaurus	0	0	0	0	?	0	1	0	1	1	1	0	1	0	0	?	1	2	1	1
Nyctiphruretus	0	0	1	0	0	1	0	0	2	1	1	1	1	0	0	2	1	1	0	0

	41									50										60
Seymouria	0	0	0	1	0	0	0	0	0	0	0	0	0	0	0	1	0	0	0	0
Limnoscelidae	1	0	0	0	0	0	0	0	1	0	0	0	0	0	0	0	0	0	1	1
Diadectidae	1	1	0	0	0	0	0	1	0	0	0	0	0	0	0	?	0	0	0	1
Synapsida	1	0	0	0	0	0	0	0	1	0	0	0	0	0	0	0&1	0	0	0&1	1
Mesosauridae	1	0	0	1	?	0	0	0	1	0	?	?	?	0	0	0	0	0	0	1
Eunotosaurus	1	?	?	?	?	?	0	0	1	?	?	?	?	0	0	1	0	0	0	1
Millerettidae	1	0	0	0	0	0	0	0	1	0	1	0	1	0	0	1	1	0	1	1
Eudibamus	?	?	?	?	?	?	1	2	2	1	1	?	?	0	0	0	1	?	0	?
Belebey	2	0	0	0	?	1	1	2	2	1	1	0	0	0	0	0	1	0	0	?
Acleistorhinus	1	0	0	0	1	0	0	0	1	1	1	1	1	1	1	0	1	1	1	1
Lanthanosuchus	1	0	0	0	1	0	0	0	1	1	1	1	0	1	1	1	1	1	1	2
Bradysaurus	2	1	1	0	1	1	0	1	1	1	1	0	1	1	0	1	1	1	0	2
Pareiasuchus	2	1	1	0	1	1	0	1	1	?	1	0&1	1	1	0	1	1	1	0	2
Scutosaurus	2	1	1	0	1	1	0	1	1	1	1	0	1	1	0	1	1	1	0	2
Procolophon	1	0	0	0	1	1	0	1	2	1	1	1	1	0	0	1	0	1	0	2
Owenetta	1	0	0	0	1	1	0	1	2	1	1	1	1	0	0	1	1	1	0	2
Barasaurus	1	0	0	0	1	1	0	1	2	?	1	1	1	0	0	1	1	1	0	2
Captorhinidae	1	0	0	1	0	0	0	0	0	0	?	?	1	0	0	1	1	0	0&1	1
Paleothyris	1	0	?	1	0	0	0	0	1	0	0	0	?	0	0	0	1	0	1	1
Araeoscelidia	1	0	?	1	0	0	0	1	0&1	0	0	0	2	0	0	1	1	0	0&1	1
Younginiformes	1	0	0	1	0	0	0	0	1	0	1	1	2	0	0	1	1	0	0	1
Macroleter	2	1	1	0	1	0	0	0	1	1	1	0	1	1	0	0	1	2	0	2
Bashkyroleter mesensis	2	1	1	0	1	0	0	0	1	0	1	0	?	1	0	1	1	2	0	?
"Bashkyroleter" bashkyricus	2	?	1	0	1	?	0	0	1	1	?	?	1	1	0	?	1	2	0	?
Nycteroleter	2	?	?	0	?	0	0	0	1	1	?	?	1	1	0	1	1	2	0	?
Emeroleter	?	?	?	0	?	0	0	0	1	0	?	?	1	1	0	1	1	2	0	?
Tokosaurus	?	?	?	0	?	?	0	0	1	0	1	0	1	?	0	0	1	2	0	2
Nyctiphruretus	1	1	1	0	1	0	0	0	1	0	0	0	1	0	0	1	1	1	0	1

	61									70										80
Seymouria	3	1	0	0	0	0	0	0	0	0	0	0	0	1	0	0	0	0	0	0
Limnoscelidae	0	0	0	1	?	0	0	0	0	0	0	0	?	?	0	0	1	1	0	0
Diadectidae	0	0	0	1	1	0	0	0	0	0	0	0	0	0	0	0	1	0	0	?
Synapsida	0	0	0	0&1	0	1	0	1	0	0&2	0	0	0	0	1	0	0&1&	0	0	0&1
Mesosauridae	0	0	0	?	?	2	?	1	0	0	?	0	0	0	1	0	?	?	0	0
Eunotosaurus	1	0	0	?	?	?	?	?	0	?	?	?	0	1	1	0	?	?	?	0
Millerettidae	0	0	0	0	0	2	1	1	0	0	0	0	0	1	1	0	?	0	0	0
Eudibamus	?	0	?	?	?	?	?	0	1	?	?	?	?	?	1	0	2	?	?	?
Belebey	?	0	0	?	?	?	0	?	?	?	0	0	?	?	1	0	2	0	1	1
Acleistorhinus	0	1	1	0	?	2	0	0	1	0	0	1	0	0	1	0	?	?	?	1
Lanthanosuchus	0	1	1	0	?	1	?	0	1	?	1	1	?	?	1	0	?	?	?	?
Bradysaurus	1	0	1	0	1	2	0	1	0	0	1	1	?	0	1	1	?	1	1	1
Pareiasuchus	1	0	1	0	?	2	?	1	0	0	1	1	?	?	1	2	2	1	1	1
Scutosaurus	1	0	1	0	1	2	0	1	0	0	1	1	0	1	1	2	2	1	1	1
Procolophon	1	0	1	0	0	2	1	1	0	1	0	1	1	1	1	0	2	1	?	1
Owenetta	1	0	1	0	?	2	?	1	0	1	0	0	?	?	1	0	2	1	1	1
Barasaurus	1	0	1	?	?	2	?	1	0	1	0	0	?	?	1	0	?	?	?	?
Captorhinidae	2	0	0	0	?	2	0	1	0	0	0	0	0	0	1	0	1	0	0	0
Paleothyris	0	0	0	?	?	2	?	1	0	0	?	0	0	0	1	0	?	?	0	0
Araeoscelidia	0	0	0	0	?	2	?	1	0	0	0	0	0	0	1	0	?	0	0	0
Younginiformes	2	0	0	0	?	2	?	1	0	0	0	0	1	1	1	0	?	?	0	?
Macroleter	1	0	1	0	1	2	0	1	0	0	1	?	2	1	1	0	2	1	1	1
Bashkyroleter mesensis	?	0	1	?	?	2	?	?	?	?	?	1	?	?	1	0	?	1	1	1
"Bashkyroleter" bashkyricus	?	0	1	?	?	?	?	?	?	?	?	1	?	?	1	0	?	1	1	1
Nycteroleter	?	?	?	?	?	?	?	?	?	0	?	1	?	?	1	0	?	?	1	1
Emeroleter	?	?	?	?	?	?	?	?	?	0	?	1	?	?	1	0	?	?	?	?
Tokosaurus	1	0	1	?	?	?	?	1	?	0	?	?	?	?	1	0	?	?	?	1
Nyctiphruretus	1	0	?	?	?	2	?	1	0	0	0	1	?	?	1	0	?	?	1	1

	81									90										100
Seymouria	0	0	0	0	0	0	0	0	0	0	0	0	0	0	0	0	0	0	0	0
Limnoscelidae	0	0	0	0	0	0	0	0	0	0	0	1	1	0	0	1	0	0	?	0
Diadectidae	0	1	0	0	0	0	0	0	0	0	0	1	1	0	0	1	0	?	?	0
Synapsida	0&1	0	0	0	?	0	0	0	0	1	0	0	0&1	1	0	1	0	0	0	0
Mesosauridae	0	1	?	1	0	0	0	?	0	1	1	0	1	0	0	1	0	0	1	0
Eunotosaurus	?	?	?	0	?	?	0	?	1	?	?	?	?	1	0	1	1	?	?	0
Millerettidae	0	1	0	0	?	0	0	0	0	1	0	0	1	1	0	1	1	?	?	0
Eudibamus	?	?	1	0	?	?	2	0	0	?	?	?	0	0	0	0	?	0	?	1
Belebey	0	1	1	0	?	0	2	1	?	?	?	?	?	0	0	?	?	?	?	?
Acleistorhinus	0	1	1	0	?	0	?	1	?	?	?	?	?	?	?	?	?	?	?	?
Lanthanosuchus	?	?	?	?	?	?	?	?	?	?	?	?	?	0	?	?	?	?	?	1
Bradysaurus	0	1	1	1	?	1	1	0	1	?	1	0	0	0	0	2	1	1	1	1
Pareiasuchus	0	1	1	1	?	1	1	0	1	?	?	?	?	0	0	2	?	?	?	1
Scutosaurus	0	1	1	1	?	1	1	0	1	?	1	0	0	0	0	?	1	1	?	1
Procolophon	1	1	1	1	1	0	1	1	0	1	1	0	?	0	1	2	1	0	0	1
Owenetta	0	1	1	1	1	0	1	1	0	?	?	0	?	0	?	2	1	0	?	1
Barasaurus	?	?	?	?	?	?	?	?	0	1	1	0	?	0	1	2	1	0	?	1
Captorhinidae	0	1	0	0	?	0	0	0	0	1	0	0	1	0	0	1	0	0	0	0
Paleothyris	0	1	?	0	?	?	0	0	0	1	1	0	1	1	0	1	0	0	0	0
Araeoscelidia	0	1	0	0	?	0	0	0&1	0	1	0	0	1	0	0	1	0	0	0	0
Younginiformes	0	1	?	0	?	0	0	0	?	?	0	0	?	1	0	1	0	1	0	1
Macroleter	1	1	1	0	?	?	?	1	0	1	1	0	1	0	0	2	1	?	?	1
Bashkyroleter mesensis	1	1	1	0	?	?	?	?	?	?	?	?	?	?	?	?	?	?	?	?
"Bashkyroleter" bashkyricus	1	1	1	0	?	?	?	?	?	?	?	?	?	?	?	?	?	?	?	?
Nycteroleter	1	?	?	0	?	?	?	1	?	?	?	?	?	?	?	?	?	?	?	?
Emeroleter	?	?	?	?	?	?	?	?	?	?	?	?	?	?	?	?	?	?	?	?
Tokosaurus	1	?	1	0	?	?	?	?	?	?	?	?	?	?	?	?	?	?	?	?
Nyctiphruretus	0	1	0	1	1	0	0	1	0	?	?	?	?	0	1	2	1	1	?	1

	101									110										120
Seymouria	0	0	0	0	0	0	0	0	0	0	0	1	0	0	0	0	0	0	0	0
Limnoscelidae	0	0	0	0	0	0	0	0	0	0	1	0	0	1	0	0	0	0	0	0
Diadectidae	0	0	0	0	0	0	0	0	0	0	1	0	0	1	1	0	0	0	0	0
Synapsida	0	1	0	0	0	0	0	0	0	0	0	0	0	0	0	0	0	0	0	0&1
Mesosauridae	0	1	0	1	0	0	0	1	0&1	0	2	1	0	0	0	0	1	1	0	1
Eunotosaurus	0	?	1	1	0	0	0	1	1	0	0	?	0	0	1	0	?	0	0	0
Millerettidae	0	1	0	1	0	0	0	1	1	0	0	?	0	0	1	?	?	0	0	1
Eudibamus	1	?	0	1	?	0	0	1	3	1	2	?	?	1	0	1	0	1	1	1
Belebey	?	?	0	?	?	?	?	1	?	?	?	?	?	0	0	?	?	?	?	1
Acleistorhinus	?	?	?	?	?	?	?	?	?	?	?	?	?	?	?	?	?	?	?	?
Lanthanosuchus	1	?	0	?	?	?	?	?	?	?	?	?	?	?	?	?	?	?	?	?
Bradysaurus	1	1	1	1	?	1	0	2	2	0	1	1	2	0	1	1	0	0	0	0
Pareiasuchus	?	2	1	1	1	1	0	?	2	1	1	1	2	0	1	1	0	0	1	0
Scutosaurus	?	2	1	1	1	1	0	2	2	0	1	1	2	0	1	1	0	0	1	0
Procolophon	1	2	1	1	1	0	0	2	0	0	0	1	0	0	1	1	0	?	0	1
Owenetta	?	1	1	1	?	0	0	1	?	1	0	1	?	0	1	1	?	?	0	1
Barasaurus	1	1	1	1	1	0	0	1	0	1	0	1	0	0	1	?	0	0	0	1
Captorhinidae	0	1&2	0	0	0	0	0	2	3	0	0	0	0	0	0	0	0	0	0	0
Paleothyris	0	1	0	0	0	0	0	1	0	0	2	0	0	0	0	0	0	0	0	1
Araeoscelidia	0	1	0	0	0	0	1	1&2	0&1	0	2	0	0	0	0	0	1	0	0	1
Younginiformes	0	1	0	1	0	0	1	1	0&1	0	0	1	0	0	1	1	1	0	0	1
Macroleter	1	1	1	1	?	0	0	1	2	0	0	1	?	0	1	1	0	0	0	1
Bashkyroleter mesensis	?	?	?	?	?	?	?	?	?	?	?	?	?	?	?	?	?	?	?	?
"Bashkyroleter" bashkyricus	?	?	?	?	?	?	?	?	?	?	?	?	?	?	?	?	?	?	?	?
Nycteroleter	?	?	?	?	?	?	?	?	?	?	?	?	?	?	?	?	?	?	?	?
Emeroleter	?	?	?	?	?	?	?	?	?	?	?	?	?	?	?	?	?	?	?	?
Tokosaurus	?	?	?	?	?	?	?	?	?	?	?	?	?	?	?	?	?	?	?	?
Nyctiphruretus	1	?	1	0	0	0	0	1	0	0	0	0	?	0	1	0	1	0	0	1

	121									130							137
Seymouria	?	0	?	?	?	?	?	0	0	0	0	0	0	0	0	0	0
Limnoscelidae	0	1	?	?	0	?	?	0	0	0	0	0	0	1	?	0	0
Diadectidae	0	1	?	0&1	0	?	0	0	0	0	0	0	0	0	0	0	0
Synapsida	0&1	2	0	0	0	0	0	0	0	0	0	0	0	1	?	0	0
Mesosauridae	0	2	0	0	1	0	0	0	0	0	1	0	0	1	?	0	0
Eunotosaurus	0	2	0	0	1	0	0	0	0	0	?	?	?	?	?	?	0
Millerettidae	1	2	0	0	1	0	?	0	1	0	1	0	0	1	?	0	0
Eudibamus	1	2	0	0	1	0	0	0	1	0	?	?	?	?	?	?	?
Belebey	?	?	?	?	?	?	?	?	?	0	1	0	0	1	?	0	0
Acleistorhinus	?	?	?	?	?	?	?	?	?	?	1	0	0	1	?	1	0
Lanthanosuchus	?	?	?	?	?	?	?	?	?	?	1	0	0	1	?	0	0
Bradysaurus	0	2	0	1	1	1	?	2	0	1	1	0	?	1	?	0	0
Pareiasuchus	0	2	0	1	1	?	?	?	?	1	1	0	?	1	?	0	0
Scutosaurus	0	2	0	1	1	1	?	2	0	1	1	0	0	1	?	0	0
Procolophon	0	2	0	0	1	1	?	1	0	0	1	0	1	0	0	0	0
Owenetta	0	?	0	?	1	?	?	?	?	0	1	0	0	0	0	0	0
Barasaurus	0	?	?	1	1	?	?	0	0	0	1	0	0	0	0	0	0
Captorhinidae	0	2	0	0	1	0	0	0	0	0	0	0	0	1	?	0	0
Paleothyris	1	2	0	0	1	0	1	0	0	0	0	0	0	1	?	0	0
Araeoscelidia	1	2	1	0	1	0	1	0	1	0	0	0	0	1	?	0	0
Younginiformes	1	2	1	0	1	0&1	1	0	1	0	0	0	0	1	?	1	0
Macroleter	0	2	0	1	1	1	0	?	0	0	1	1	0	0	0	0	0
Bashkyroleter mesensis	?	?	?	?	?	?	?	?	?	?	0	0	1	0	1	0	1
"Bashkyroleter" bashkyricus	?	?	?	?	?	?	?	?	?	?	0	0	1	0	1	?	0
Nycteroleter	?	?	?	?	?	?	?	?	?	?	0	0	0	0	1	0	0
Emeroleter	?	?	?	?	?	?	?	?	?	?	1	0	0	0	1	0	1
Tokosaurus	?	?	?	?	?	?	?	?	?	?	1	1	0	0	0	0	0
Nyctiphruretus	0	2	1	0	0	0	0	?	0	0	1	0	0	1	?	1	0
