# Supplementary figures and images for: Impedance-Matching Hearing in Paleozoic Reptiles: Evidence of Advanced Sensory Perception at an Early Stage of Amniote Evolution
Source: PLoS One. 2007 Sep 12;2(9):e889. doi: 10.1371/journal.pone.0000889 (PMC1964539; doi:10.1371/journal.pone.0000889)

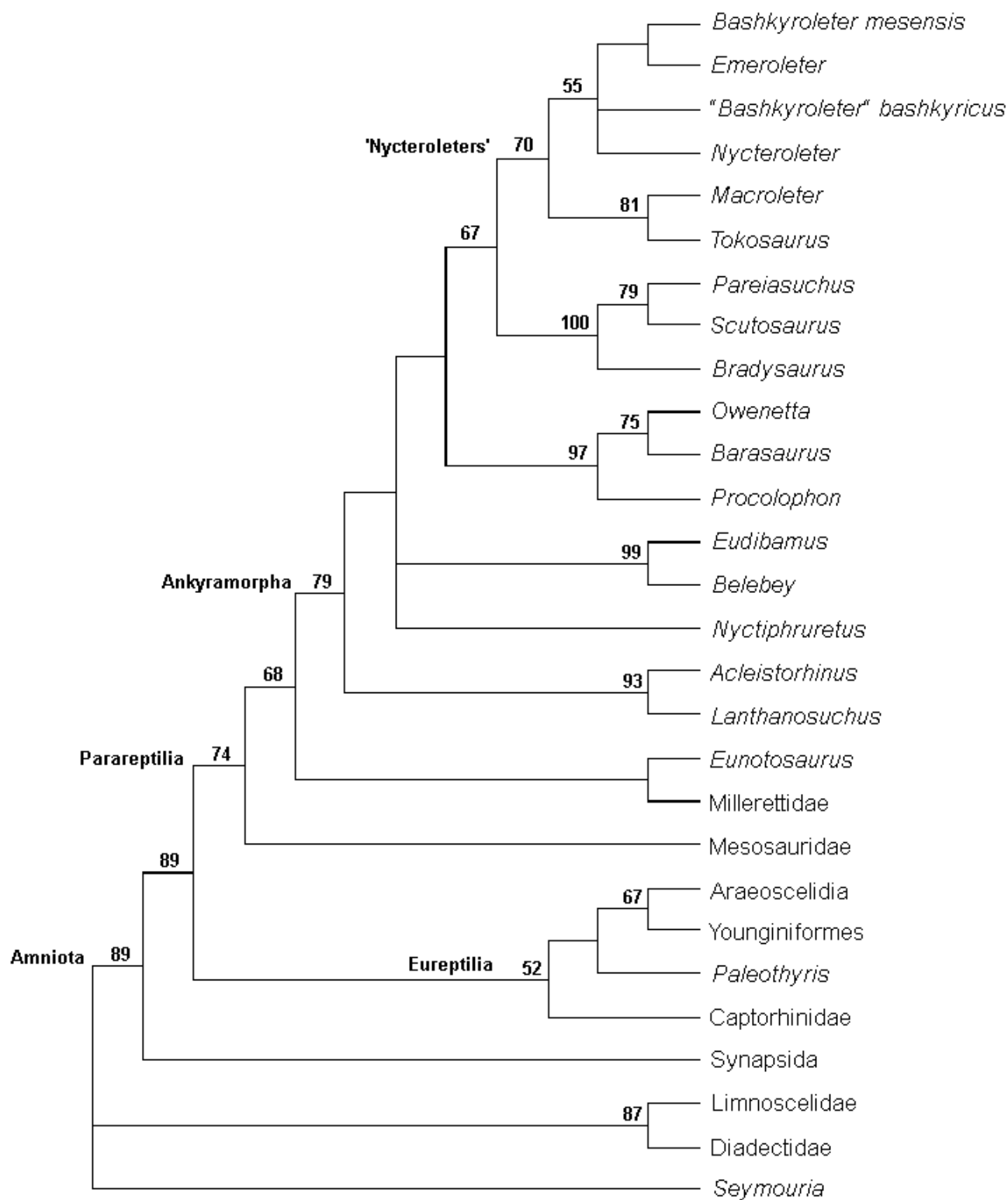

Supplement: Figure S1 — Strict consensus tree of the phylogenetic analysis with bootstrap values (1000 replicates.) (0.01 MB PDF) [file pone.0000889.s004.pdf]
